# A Secure Lightweight Three-Factor Authentication Scheme for IoT in Cloud Computing Environment

**DOI:** 10.3390/s19163598

**Published:** 2019-08-19

**Authors:** SungJin Yu, KiSung Park, YoungHo Park

**Affiliations:** School of Electronics Engineering, Kyungpook National University, Daegu 41566, Korea

**Keywords:** authentication, cloud computing, internet of things, formal security analysis, Burrows–Abadi–Needham logic, AVISPA

## Abstract

With the development of cloud computing and communication technology, users can access the internet of things (IoT) services provided in various environments, including smart home, smart factory, and smart healthcare. However, a user is insecure various types of attacks, because sensitive information is often transmitted via an open channel. Therefore, secure authentication schemes are essential to provide IoT services for legal users. In 2019, Pelaez et al. presented a lightweight IoT-based authentication scheme in cloud computing environment. However, we prove that Pelaez et al.’s scheme cannot prevent various types of attacks such as impersonation, session key disclosure, and replay attacks and cannot provide mutual authentication and anonymity. In this paper, we present a secure and lightweight three-factor authentication scheme for IoT in cloud computing environment to resolve these security problems. The proposed scheme can withstand various attacks and provide secure mutual authentication and anonymity by utilizing secret parameters and biometric. We also show that our scheme achieves secure mutual authentication using Burrows–Abadi–Needham logic analysis. Furthermore, we demonstrate that our scheme resists replay and man-in-the-middle attacks usingthe automated validation of internet security protocols and applications (AVISPA) simulation tool. Finally, we compare the performance and the security features of the proposed scheme with some existing schemes. Consequently, we provide better safety and efficiency than related schemes and the proposed scheme is suitable for practical IoT-based cloud computing environment.

## 1. Introduction

With the recent advances in wireless sensor networks and embedded technologies, internet of things (IoT) connects objects and shares various useful data with internet through resource-constrained devices to provide convenient services for users such as smart home, healthcare, vehicle to everything and smart gird. However, a single server environment also is inefficient for IoT because an ocean of data is generated by resource-constrained devices such as microsensor, RFID tag and smart cards.

Cloud computing is a distributed computing mechanism for a large-scale data and allows sharing resources among all of the servers and users. The cloud computing provides five essential characteristics: *on-demand self-services*, *ubiquitous network access*, *rapid elasticity*, *measured service* and *resource pooling* [1,2]. *On-demand self-service* handles cloud services without human interaction and *ubiquitous network access* controls access service using standard protocols. *Rapid elasticity* and *measured service* optimize the resource usage. *Resource pooling* provides cloud service using homogeneous infrastructure among service users. The cloud computing deals with an ocean of data generated by devices and sensors and provides data managing service for users through these essential characteristics.

However, these services are vulnerable to potential attacks by malicious adversaries because they are provided through an open channel, including sensitive data of legitimate user about location, health, payment, etc. Therefore, a secure and efficient authentication for IoT environment has become essential security requirements to provide useful services to user.

In 1981, Lamport [3] proposed one factor user authentication scheme using passwords to ensure user’s privacy. However, security of the password based authentication scheme is easily broken because its security only relies on the passwords. In 2002, Chien et al. proposed two factor authentication scheme to overcome this security flaw using password and smart cards. However, their scheme is vulnerable to smart card stolen attack as the data stored in smart cards can be extracted by power analysis attacks [4]. When a malicious adversary obtains smart cards and password, they can perform various attacks such as impersonation, replay and insider attacks. To overcome the above-mentioned security weaknesses, three-factor authentication schemes have been proposed [5,6,7]. Biometrics (e.g., face, retina, fingerprint, iris, etc.) have several important characteristics: they cannot be lost or forgotten; they are hard to forge, copy, share or distribute; and they are difficult to guess.

In 2019, Pelaez et al. [8] demonstrated that the previous scheme is vulnerable to insider, off-line guessing and disclosure attacks and proposed enhanced IoT-based authentication scheme in cloud computing environment. This paper demonstrates that Pelaez et al.’s scheme does not withstand impersonation, session key disclosure and replay attacks. We also show that their scheme does not achieve secure mutual authentication and anonymity. Moreover, we propose a secure and lightweight three-factor authentication scheme for IoT in cloud computing environment to resolve these security weaknesses, considering computational costs.

### 1.1. Adversary Model

We present the Dolev–Yao (DY) model [9] to evaluate security of ours and previous schemes, which is widely accepted as security threat model. The detailed description of the DY model is as below:

- A malicious adversary can modify, intercept, delete or insert the transmitted messages via an open channel. A malicious adversary can obtain or steal the smart card of legitimate user and can extract the data stored in the smart card by using power-analysis [4].
- A malicious adversary can perform various attacks such as man-in-the-middle (MITM), replay, impersonation, and session key disclosure attack [10,11].

### 1.2. Our Contributions

Our contributions in this paper are as follows.

- We demonstrate that Pelaez et al.’s scheme is not secure against various attacks such as impersonation, session key disclosure and replay attacks and does not achieve secure mutual authentication and anonymity.
- We propose a secure and lightweight three-factor authentication scheme for IoT in cloud computing environment to address the security shortcomings of Pelaez et al.’s scheme. The proposed scheme withstands impersonation, session key disclosure, and replay attacks and achieve secure mutual authentication and anonymity. Moreover, the proposed scheme is more efficient than Pelaez et al.’s scheme because it utilizes only bitwise exclusive or (XOR) and hash operations.
- We prove that the proposed scheme provides secure mutual authentication using the Burrows–Abadi–Needham (BAN) logic [12] and perform an informal security analysis to prove that our scheme is secure against various attacks such as MITM, impersonation, replay and session key disclosure attacks. Furthermore, we compare the security properties and performance of proposed protocol with other related schemes.
- We perform a formal security analysis using the automated validation of internet security protocols and applications (AVISPA) simulation tool to prove that the proposed protocol resists the MITM and replay attacks.

### 1.3. Organization

We introduce the related works and review Pelaez et al.’s scheme in Section 2 and Section 3. In Section 4 and Section 5, we cryptanalyze Pelaez et al.’s scheme and propose a lightweight IoT-based three-factor authentication scheme in cloud computing environment to enhance the security shortcomings of Pelaez et al.’s scheme. Section 6 and Section 7 prove the security of proposed scheme and present the simulation analysis using AVISPA. In Section 8, we compare the security properties and performances of proposed protocol with other related schemes. Finally, Section 9 concludes the paper.

## 2. Related Works

In last few decades, numerous authentication and key agreement schemes have been proposed to ensure privacy of user, considering resource-constrained environments such as wireless sensor networks, global mobility networks and vehicular networks [3,13,14,15,16,17,18,19]. In 1981, Lamport [3] firstly proposed a lightweight password based user authentication scheme to provide secure communication. However, Lamport’s scheme has low security level because its security only relies on passwords. In 2002, Chien et al. [13] presented a two-factor user authentication protocol using smart card and password to resolve this problem. Unfortunately, the two-factor authentication schemes using password and smart cards cannot ensure user’s privacy [13,14,15,16,17,18,19], when the data stored in token (e.g., smart card, mobile device, etc.) are compromised.

Later, several authentication and key agreement schemes for IoT have been presented in various fields [20,21,22]. However, these environments are not suitable for IoT because it cannot handle a large number of data. In 2019, Zhou et al. [23] presented a lightweight IoT-based authentication scheme in cloud computing environment to overcome this issue. Zhou et al. claimed that their scheme can prevent various attacks such as insider, forgery and tracking attacks and provide secure mutual authentication and session key security. However, in 2019, Pelaez et al. [8] pointed out that Zhou et al.’s scheme [23] cannot withstand insider, off-line guessing and session key disclosure attacks and provide secure mutual authentication. To resolve these security problems, Pelaez et al. [8] presented a lightweight IoT-based authentication scheme in cloud computing environment. They also claimed that their scheme is secure against off-line password guessing, insider, impersonation and replay attacks.

## 3. Review of Pelaez et al.’s Scheme

We briefly review Pelaez et al.’s IoT based authentication scheme in cloud computing environment. Their scheme comprises of three processes: registration, authentication, and password change. These processes are presented as below (for details, see [8]).

### 3.1. User Registration Process

**Figure 1:**
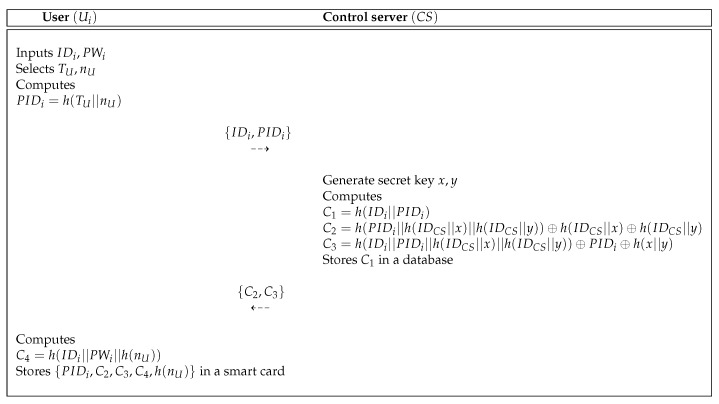
User registration process of the Pelaez et al.’s scheme [8].

In Pelaez et al.’s scheme, a new user $U_{i}$ is registered from control server CS via a secure channel. Figure 1 shows the user registration process of Pelaez et al.’s scheme. In Figure 1, $U_{i}$ sends the registration request to CS and then CS issues the smart cards.

### 3.2. Cloud Server Registration Process

**Figure 2:**
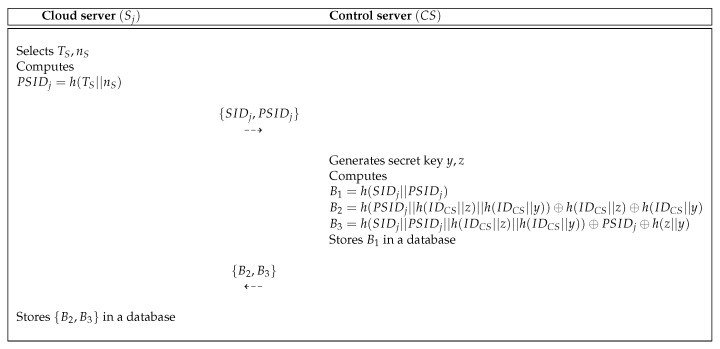
Cloud server registration process of the Pelaez et al.’s scheme [8].

In Pelaez et al.’s scheme, a cloud server $S_{j}$ is registered from control server CS via a secure channel. Figure 2 shows the cloud server registration process of the Pelaez et al.’s scheme. In Figure 2, $S_{j}$ sends the registration request to CS and then CS sends parameters $B_{2}$ and $B_{3}$ to $S_{j}$.

### 3.3. Login Process

**Figure 3:**
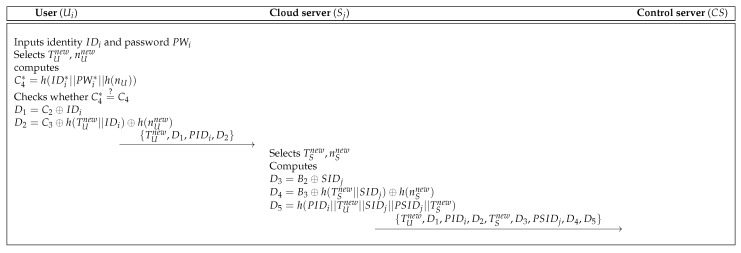
Login process of the Pelaez et al.’s scheme [8].

When $U_{i}$ wants to access the service, $U_{i}$ firstly sends login request message to $S_{j}$. In Figure 3, $U_{i}$ sends login request messages $\left\{T_{U}^{new},D_{1},PID_{i},D_{2}\right\}$ to $S_{j}$, and then $S_{j}$ sends the messages $\left\{T_{U}^{new},D_{1},PID_{i},D_{2},T_{S}^{new},D_{3},PSID_{j},D_{4},D_{5}\right\}$ to CS in order to check validation of $U_{i}$.

### 3.4. Authentication Process

**Figure 4:**
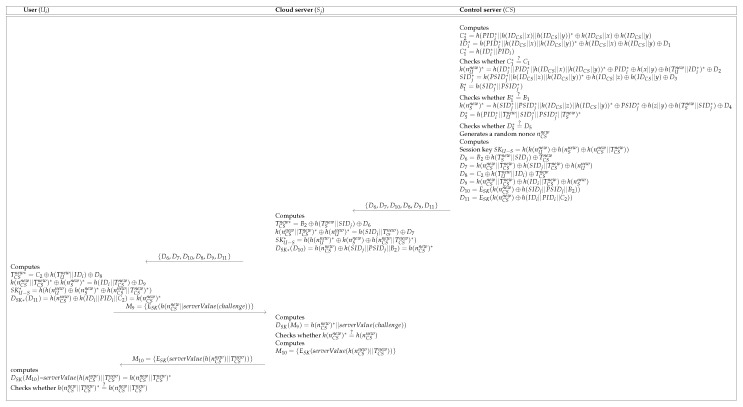
Authentication process of the Pelaez et al.’s scheme [8].

After finishing the login process, $U_{i}$, $S_{j}$ and CS perform mutual authentication with each entity, and then $U_{i}$ and $S_{j}$ can share the session key $SK_{U-S}$. Figure 4 shows the authentication process of the Pelaez et al.’s scheme.

## 4. Cryptanalysis of Pelaez et al.’s Scheme

In this section, we demonstrate that Pelaez et al.’s scheme does not resist replay, session key disclosure and impersonation attacks and show that their scheme does not achieve secure mutual authentication and anonymity.

### 4.1. Impersonation Attack

The impersonation attack is that a malicious adversary try to impersonate as a legitimate user. When a malicious adversary $U_{MA}$ may attempt to impersonate a legal user, $U_{MA}$ can easily generate the login request message of $U_{i}$. According to Section 1.1, $U_{MA}$ can obtain smart card of $U_{i}$ and can extract the data $\left\{PID_{i},C_{2},C_{3},C_{4},h\left(n_{U}\right)\right\}$ stored in smart card. Furthermore, $U_{MA}$ intercepts the message transmitted via an open channel. Finally, $U_{MA}$ performs the impersonation attack as below:

**Step** **1:** A malicious adversary $U_{MA}$ can compute real identity $ID_{i}=C_{2}⊕D_{1}$ of legitimate user $U_{i}$ and $h\left(n_{U}^{new}\right)=D_{2}⊕C_{3}⊕h(T_{MA}^{new}||ID_{i})$. Then, $U_{MA}$ generates timestamp $T_{MA}^{new}$ and random nonce $n_{MA}^{new}$, computes $D_{2MA}=C_{3}⊕h(T_{MA}^{new}||ID_{i})⊕h\left(n_{MA}^{new}\right)$, and sends $\left\{T_{MA}^{new},D_{1},PID_{i},D_{2MA}\right\}$ to the $S_{j}$.
**Step** **2:** Upon getting the message from $U_{MA}$, the $S_{j}$ generates random nonces $T_{S}^{new}$ and $n_{S}^{new}$ and computes $D_{3}=B_{2}⊕SID_{j}$, $D_{4}=B_{3}⊕h(T_{S}^{new}||SID_{j})⊕h\left(n_{S}^{new}\right)$ and $D_{5}=h(PID_{i}\left|\right|T_{MA}^{new}||SID_{j}||PSID_{j}\left|\right|T_{S}^{new})$. Then, the $S_{j}$ sends $\left\{T_{MA}^{new},D_{1},PID_{i},D_{2MA},T_{S}^{new},D_{3},PSID_{j},D_{4},D_{5}\right\}$ to the CS.
**Step** **3:** Upon getting the message from $S_{j}$, the CS computes $C_{2}^{*}\;=\;h(PID_{i}^{*}||h(ID_{CS}||x)||h(ID_{CS}{\left||y)\right)}^{*}⊕h(ID_{CS}||x)⊕h(ID_{CS}\left||y\right)$, $ID_{i}^{*}=h(PID_{i}^{*}||h(ID_{CS}||x)||h(ID_{CS}{\left||y)\right)}^{*}⊕h(ID_{CS}||x)⊕h(ID_{CS}\left||y\right)⊕D_{1}$ and $C_{1}^{*}=h(ID_{i}^{*}||PID_{i})$. Then, the CS checks whether $C_{1}^{*}\overset{?}{=}C_{1}$. If it is valid, the CS authenticates $U_{MA}$. Then, the CS computes $h{\left(n_{MA}^{new}\right)}^{*}=h(ID_{i}^{*}||PID_{i}^{*}||h(ID_{CS}||x)||h(ID_{CS}{\left||y)\right)}^{*}⊕PID_{i}^{*}⊕h(x||y)⊕h(T_{MA}^{new}||ID_{i}^{*}{)}^{*}⊕D_{2}$. After that, the CS computes $SID_{j}^{*}=h(PSID_{j}^{*}||h(ID_{CS}||z)||h(ID_{CS}{\left||y)\right)}^{*}⊕h(ID_{CS}||z)⊕h(ID_{CS}\left||y\right)⊕D_{3}$ and $B_{1}^{*}=h(SID_{j}^{*}||PSID_{j}^{*})$. Then, the CS checks whether $B_{1}^{*}\overset{?}{=}B_{1}$. If it is valid, the CS authenticate $S_{j}$. After that, the CS recovers $h{\left(n_{S}^{new}\right)}^{*}=h(SID_{j}^{*}||PSID_{j}^{*}||h(ID_{CS}||z)||h(ID_{CS}{\left||y)\right)}^{*}⊕PSID_{j}^{*}⊕h(z||y)⊕h(T_{S}^{new}||SID_{j}^{*})⊕D_{4}$. Then, the CS computes $D_{5}^{*}=h(PID_{i}^{*}\left|\right|T_{MA}^{new}||SID_{j}^{*}||PSID_{j}^{*}\left|\right|T_{S}^{new}{)}^{*}$ and checks whether $D_{5}^{*}\overset{?}{=}D_{5}$. If it is valid, the CS have evidence of the connection attempt between $U_{MA}$ and $S_{j}$. To key agreement and mutual authentication, the CS generates a random nonce $n_{CS}^{new}$ and computes the session key $SK_{MA-S}=h(h\left(n_{MA}^{new}\right)⊕h\left(n_{S}^{new}\right)⊕h(n_{CS}^{new}\left|\right|T_{CS}^{new}))$. Then, the CS computes $D_{6}=B_{2}⊕h(T_{S}^{new}||SID_{j})⊕T_{CS}^{new}$, $D_{7MA}=h(n_{CS}^{new}\left|\right|T_{CS}^{new})⊕h(SID_{j}\left|\right|T_{CS}^{new})⊕h\left(n_{MA}^{new}\right)$, $D_{8MA}=C_{2}⊕h(T_{MA}^{new}||ID_{i})⊕T_{CS}^{new}$, $D_{9}=h(n_{CS}^{new}\left|\right|T_{CS}^{new})⊕h(ID_{i}\left|\right|T_{CS}^{new})⊕h\left(n_{S}^{new}\right)$, $D_{10MA}=E_{SK}(h\left(n_{CS}^{new}\right)⊕h(SID_{j}||PSID_{j}\left|\right|B_{2}))$ and $D_{11MA}=E_{SK}(h\left(n_{CS}^{new}\right)⊕h(ID_{i}||PID_{i}\left|\right|C_{2}))$. Finally, the CS sends $\left\{D_{6},D_{7MA},D_{10MA},D_{8MA},D_{9},D_{11MA}\right\}$ to the $S_{j}$.
**Step** **4:** Upon getting the message from CS, the $S_{j}$ computes $T_{CS}^{new*}=B_{2}⊕h(T_{S}^{new}||SID_{j})⊕D_{6}$, $h(n_{CS}^{new}\left|\right|T_{CS}^{new}{)}^{*}⊕h{\left(n_{MA}^{new}\right)}^{*}=h(SID_{j}\left|\right|T_{cs}^{new})⊕D_{7MA}$, $SK_{U-S}^{*}=h(h{\left(n_{MA}^{new}\right)}^{*}⊕h\left(n_{S}^{new}\right)⊕h(n_{CS}^{new}\left|\right|T_{CS}^{new}{)}^{*})$ and decrypts $D_{SK*}\left(D_{10MA}\right)=h\left(n_{CS}^{new}\right)⊕h(SID_{j}||PSID_{j}\left|\right|B_{2})=h{\left(n_{CS}^{new}\right)}^{*}$. After that, the $S_{j}$ sends $\left\{D_{6},D_{7MA},D_{10MA},D_{8MA},D_{9},D_{11MA}\right\}$ to the $U_{MA}$.
**Step** **5:** Upon getting the messages from $S_{j}$, the $U_{MA}$ computes $T_{CS}^{new*}=C_{2}⊕h(T_{MA}^{new}||ID_{i})⊕D_{8MA}$, $h(n_{CS}^{new}\left|\right|T_{CS}^{new}{)}^{*}⊕h{\left(n_{S}^{new}\right)}^{*}=h(ID_{i}\left|\right|T_{CS}^{new})⊕D_{9}$, $SK_{MA-S}^{*}=h(h\left(n_{U}^{new}\right)⊕h{\left(n_{S}^{new}\right)}^{*}⊕h(n_{CS}^{new}\left|\right|T_{CS}^{new}{)}^{*})$ and decrypts $D_{SK*}\left(D_{11MA}\right)=h\left(n_{CS}^{new}\right)⊕h(ID_{i}||PID_{i}\left|\right|C_{2})=h{\left(n_{CS}^{new}\right)}^{*}$. For mutual authentication with $S_{j}$, the $U_{MA}$ computes $M_{9MA}=$$\{E_{SK}(h(n_{CS}^{new}\left||serverValue\left(challenge\right)))\right\}$ and sends $M_{9MA}$ to the $S_{j}$.
**Step** **6:** Upon getting the messages from $U_{MA}$, the $S_{j}$ computes $D_{SK}\left(M_{9MA}\right)\;=\;h{\left(n_{CS}^{new}\right)}^{*}||serverValue\left(challenge\right))$ and checks whether $h{\left(n_{CS}^{new}\right)}^{*}\overset{?}{=}h\left(n_{CS}^{new}\right)$. Finally, the $S_{j}$ computes $M_{10MA}=$$\{E_{SK}(serverValue(h\left(n_{CS}^{new}\right)\left|\right|T_{CS}^{new}))\}$ and sends $M_{10MA}$ to the $U_{MA}$.
**Step** **7:** Upon getting the messages from $S_{j}$, the $U_{MA}$ computes $D_{SK}\left(M_{10MA}\right)$ = $serverValue(h\left(n_{CS}^{new}\right)||T_{CS}^{new})=$$h(n_{CS}^{new}\left|\right|T_{CS}^{new}{)}^{*}$ and checks whether $h(n_{CS}^{new}\left|\right|T_{CS}^{new}{)}^{*}\overset{?}{=}h(n_{CS}^{new}\left|\right|T_{CS}^{new})$.

$U_{MA}$ can successfully generates the login request message and session key between $U_{MA}$ and $S_{j}$. As a result, we show that Pelaez et al.’s scheme cannot withstand impersonation attack.

### 4.2. Session Key Disclosure Attack

The session key disclosure attack is that a malicious adversary can obtain the session key between $U_{i}$ and $S_{j}$. Pelaez et al. claimed that their scheme can ensure security of session key because a malicious adversary cannot obtain random nonce $n_{U}^{new}$, $n_{S}^{new}$, $n_{CS}^{new}$ and current timestamp $T_{CS}^{new}$. However, according to Section 1.1, a malicious adversary $U_{MA}$ can extract the data $\left\{PID_{i},C_{2},C_{3},C_{4},h\left(n_{U}\right)\right\}$ stored in the smart card and can obtain the transmitted messages $D_{1},D_{2},T_{U}^{new},D_{8},D_{9}$ via an open channel. Therefore, a malicious adversary $U_{MA}$ can easily compute session key $SK_{U-S}^{*}=h(h{\left(n_{U}^{new}\right)}^{*}⊕h\left(n_{S}^{new}\right)⊕h(n_{CS}^{new}\left|\right|T_{CS}^{new}{)}^{*})$.

### 4.3. Replay Attack

Replay attack is that a malicious adversary try to obtain sensitive messages of user using the messages transmitted in previous and current session. Pelaez et al. claimed that their scheme can resist replay attack because a malicious adversary $U_{MA}$ cannot obtain random nonce and timestamp. However, $U_{MA}$ can calculate the random nonce and timestamp of legitimate user correctly. According to 4.1, $U_{MA}$ also impersonates a legitimate user $U_{i}$. Therefore, $U_{MA}$ can obtain $n_{U}^{new}$, $n_{S}^{new}$ and $n_{CS}^{new}$ and timestamp $T_{U}^{new},$$T_{S}^{new}$ and $T_{CS}^{new}$. As a result, Pelaez et al.’s scheme does not withstand replay attack.

### 4.4. Mutual Authentication

Pelaez et al claimed that their protocol allows secure mutual authentication among the user $U_{i}$, the cloud server $S_{j}$, and the control server CS. However, according to Section 3.1, their protocol does not withstand to impersonation attack, as a malicious adversary $U_{MA}$ can successfully generate authentication request message $D_{2}=C_{3}⊕h(T_{U}^{new}||ID_{i})⊕h\left(n_{U}^{new}\right)$. Therefore, Pelaez et al.’s scheme does not achieve secure mutual authentication.

### 4.5. Anonymity

Pelaez et al claimed that a malicious adversary $U_{MA}$ cannot obtain the real identity $ID_{i}$ of legitimate user. However, according to Section 1.1, a malicious adversary $U_{MA}$ can extract the secret parameter $C_{2}$ stored in the smart card and can intercept the transmitted message $D_{1}$ via an open channel. $U_{MA}$ can also compute $ID_{i}=C_{2}⊕D_{1}$ and easily obtain real identity of legitimate user $U_{i}$. Therefore, Pelaez et al.’s scheme does not guarantee anonymity.

## 5. Proposed Scheme

In this section, we propose a secure and lightweight three-factor authentication scheme for IoT in cloud computing environment to enhance security drawbacks of Pelaez et al.’s scheme. The proposed scheme consists of three processes: registration, login and authentication, and password change. The details of each process are presented below.

### 5.1. User Registration Process

**Figure 5:**
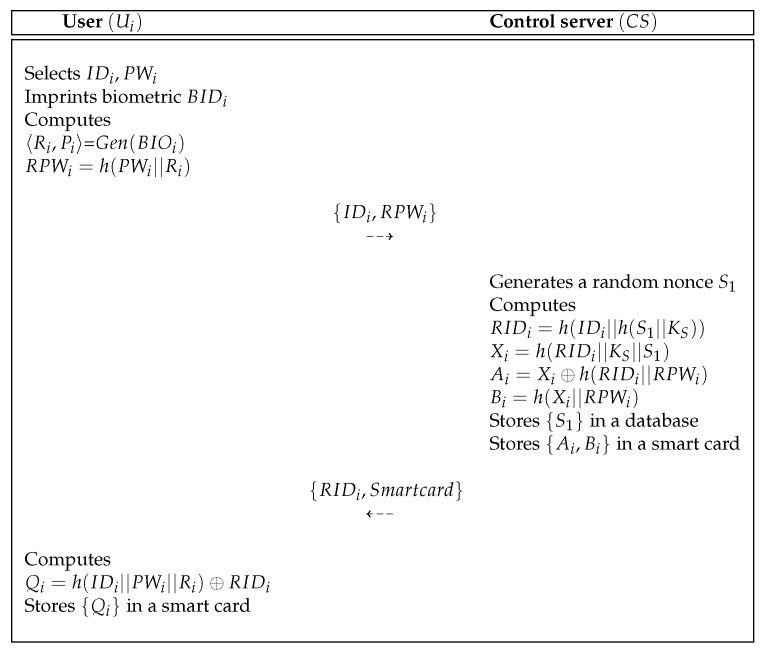
User registration process of the proposed scheme.

A new user $U_{i}$ who requests the use of the IoT services must register with control server CS. Figure 5 shows the user registration process of proposed scheme and the detailed processes are as below.

**Step** **1:** The $U_{i}$ selects $ID_{i}$ and $PW_{i}$ and imprints biometric $BIO_{i}$. After that, $U_{i}$ computes $\langle R_{i},P_{i}\rangle$=$Gen(BIO_{i})$, $RPW_{i}=h(PW_{i}\left|\right|R_{i})$ and sends messages $\left\{ID_{i},RPW_{i}\right\}$ to control server CS via a secure channel.
**Step** **2:** After getting the messages from $U_{i}$, the CS generates a random nonce $S_{1}$ and computes $RID_{i}\;=\;h(ID_{i}||h(S_{1}\left|\right|K_{S}))$, $X_{i}=h(RID_{i}\left|\right|K_{S}\left|\right|S_{1})$, $A_{i}=X_{i}⊕h(RID_{i}||RPW_{i})$, and $B_{i}\;=\;h(X_{i}||RPW_{i})$. Then, the CS stores $\left\{S_{1}\right\}$, $\left\{A_{i},B_{i}\right\}$ in a database and smart card, respectively. The CS sends $\left\{RID_{i}\right\}$ and issues smart card to $U_{i}$ via a secure channel.
**Step** **3:** After getting the message and smart card from CS, the $U_{i}$ computes $Q_{i}=h(ID_{i}||PW_{i}\left|\right|R_{i})⊕RID_{i}$ and stores $\left\{Q_{i}\right\}$ in a smart card SC.

### 5.2. Cloud Server Registration Process

**Figure 6:**
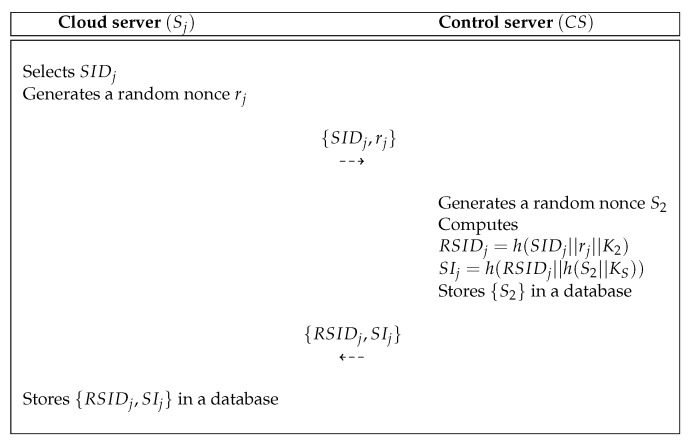
Cloud server registration process of the proposed scheme.

A cloud server $S_{j}$ must register with the control server CS to provide IoT service to the users. Figure 6 shows the cloud server registration process of proposed scheme and the detailed processes are as below.

**Step** **1:** The cloud server $S_{j}$ selects $SID_{j}$ and generates a random nonce $r_{j}$. After that, the $S_{j}$ sends messages $\left\{SID_{j},r_{j}\right\}$ to the CS via a secure channel.
**Step** **2:** After getting the messages, the CS generates a random nonce $S_{2}$ and computes $RSID_{j}\;=\;h(SID_{j}\left|\right|r_{j}\left|\right|K_{S})$ and $SI_{j}=h(RSID_{j}||h(S_{2}\left|\right|K_{S}))$. Then, the CS stores $\left\{S_{2}\right\}$ in a database and sends messages $\left\{RSID_{j},SI_{j}\right\}$ to the $S_{j}$ via a secure channel.
**Step** **3:** After getting the messages, the $S_{j}$ stores $\left\{RSID_{j},SI_{j}\right\}$ in a database.

### 5.3. Login and Authentication Process

**Figure 7:**
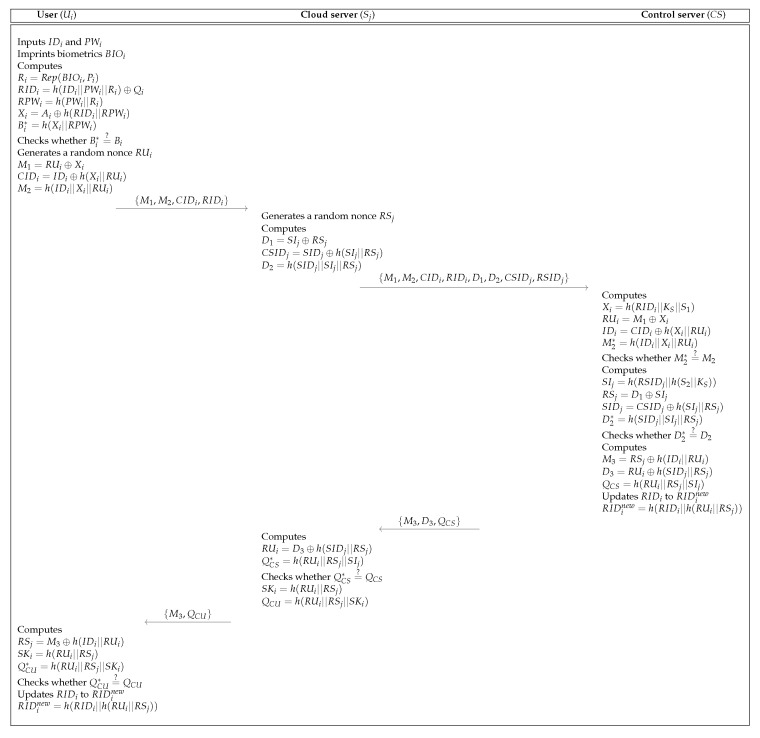
Login and authentication process of the proposed scheme.

A user $U_{i}$ who requests access to IoT service must send a login request message to the CS. Figure 7 shows the login and authentication process of the proposed scheme. The detailed process is as below.

**Step** **1:** The $U_{i}$ inputs $ID_{i}$, $PW_{i}$ and imprints biometric $BID_{i}$. Then, the $U_{i}$ calculates $R_{i}\;=\;Rep\left(BIO_{i},P_{i}\right)$, $RID_{i}=h(ID_{i}||PW_{i}\left|\right|R_{i})⊕Q_{i}$, $RPW_{i}=h(PW_{i}\left|\right|R_{i})$, $X_{i}=A_{i}⊕h(RID_{i}||RPW_{i})$ and $B_{i}^{*}=h(X_{i}||RPW_{i})$. The $U_{i}$ checks whether $B_{i}^{*}\overset{?}{=}B_{i}$. If it is correct, the $U_{i}$ generates a random nonce $RU_{i}$. After that, the $U_{i}$ computes $M_{1}=RU_{i}⊕X_{i}$, $CID_{i}=ID_{i}⊕h(X_{i}||RU_{i})$ and $M_{2}=h(ID_{i}\left|\right|X_{i}||RU_{i})$ and sends login request messages $\left\{M_{1},M_{2},CID_{i},RID_{i}\right\}$ to the $S_{j}$ via an open channel.
**Step** **2:** Upon getting the messages from the $U_{i}$, the $S_{j}$ generates a random nonce $RS_{j}$ and computes $D_{1}=SI_{j}⊕RS_{j}$, $CSID_{j}=SID_{j}⊕h(SI_{j}||RS_{j})$ and $D_{2}=h(SID_{j}||SI_{j}||RS_{j})$. Then, the $S_{j}$ sends the messages $\left\{M_{1},M_{2},CID_{i},RID_{i},D_{1},D_{2},CSID_{j},RSID_{j}\right\}$ to the CS via an open channel.
**Step** **3:** Upon getting the messages from the $S_{j}$, the CS computes $X_{i}=h(RID_{i}\left|\right|K_{S}\left|\right|S_{1})$, $RU_{i}\;=\;M_{1}⊕X_{i}$, $ID_{i}=CID_{i}⊕h(X_{i}||RU_{i})$, and $M_{2}^{*}=h(ID_{i}\left|\right|X_{i}||RU_{i})$ and checks whether $M_{2}^{*}\overset{?}{=}M_{2}$. If it is correct, the CS computes $SI_{j}=h(RSID_{j}||h(S_{2}\left|\right|K_{S}))$, $RS_{j}=h\left(D_{1}\right)⊕SI_{j}$, $SID_{j}=CSID_{j}⊕h(SI_{j}||RS_{j})$, and $D_{2}^{*}=h(SID_{j}||SI_{j}||RS_{j})$ and checks whether $D_{2}^{*}\overset{?}{=}D_{2}$. If it is valid, the CS computes $M_{3}=RS_{j}⊕h(ID_{i}||RU_{i})$, $D_{3}=RU_{i}⊕h(SID_{j}||RS_{j})$ and $Q_{CS}=h(RU_{i}||RS_{j}||SI_{j})$. Then, the CS updates $RID_{i}$ to $RID_{i}^{new}$ and replaces $\left\{RID_{i}\right\}$ with $\left\{RID_{i}^{new}\right\}$. Finally, the CS sends messages $\left\{M_{3},D_{3},Q_{CS}\right\}$ to the $S_{j}$.
**Step** **4:** Upon getting the messages from the CS, the $S_{j}$ computes $RU_{i}=D_{3}⊕h(SID_{j}||RS_{j})$ and $Q_{CS}^{*}=h(RU_{i}||RS_{j}||SI_{j})$ and checks whether $Q_{CS}^{*}\overset{?}{=}Q_{CS}$. If it is valid, the $S_{j}$ computes $SK_{i}\;=\;h(RU_{i}||RS_{j})$ and $Q_{CU}=h(RU_{i}||RS_{j}||SK_{i})$ and sends messages $\left\{M_{3},Q_{CU}\right\}$ to the $U_{i}$.
**Step** **5:** Upon getting the messages from the $S_{j}$, the $U_{i}$ computes $RS_{j}=M_{3}⊕h(ID_{i}||RU_{i})$, $SK_{i}\;=\;h(RU_{i}||RS_{j})$ and $Q_{CU}^{*}=h(RU_{i}||RS_{j}||SK_{i})$ and checks whether $Q_{CU}^{*}\overset{?}{=}Q_{CU}$. If it is correct, the $U_{i}$ computes $RID_{i}^{new}=h(RID_{i}||h(RU_{i}||RS_{j}))$ and $RID_{i}$ to $RID_{i}^{new}$. After that, the smart card updates $A_{i}^{new}=X_{i}⊕h(RID_{i}^{new}\left|\right|RPW_{i}$) and $Q_{i}^{new}=h(ID_{i}||PW_{i}\left|\right|R_{i})⊕RID_{i}^{new}$ and replaces $\left\{A_{i},Q_{i}\right\}$ with $\left\{A_{i}^{new},Q_{i}^{new}\right\}$. As a result, the $U_{i}$, $S_{j}$ and CS achieve the mutual authentication successfully.

### 5.4. Password Change Process

**Figure 8:**
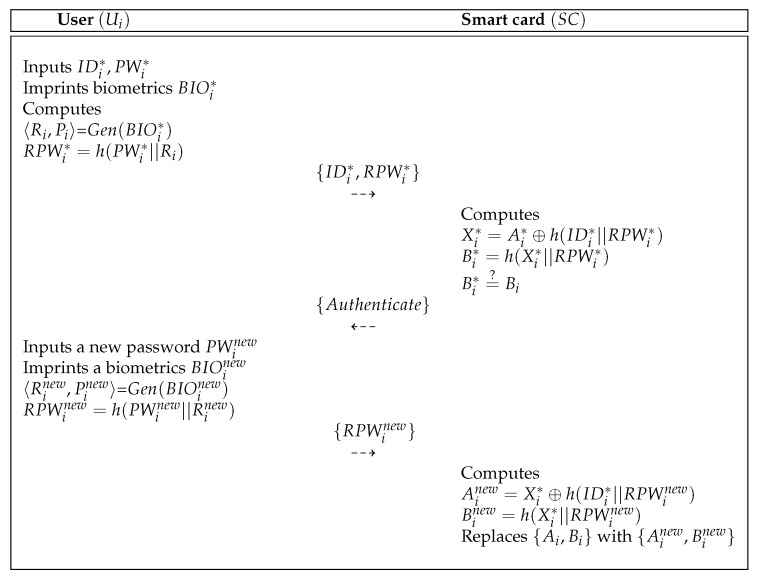
Password change process of the proposed scheme.

When $U_{i}$ wants to update his/her password, the $U_{i}$ can freely update their password in the proposed scheme. Figure 8 shows the password change process of the proposed scheme. The detailed process is as below.

**Step** **1:** The $U_{i}$ chooses $ID_{i}^{*}$, $PW_{i}^{*}$ and imprints biometrics $BIO_{i}^{*}$. Then, the $U_{i}$ calculates $\langle R_{i},P_{i}\rangle$=$Gen(BIO_{i}^{*})$, $RPW_{i}^{*}=h(PW_{MU}\left|\right|R_{i})$ and sends $\left\{ID_{MU}^{*},RPW_{i}^{*}\right\}$ to the smart card SC.
**Step** **2:** After getting the message from $U_{i}$, the SC computes $X_{i}^{*}=A_{i}^{*}⊕h(ID_{i}^{*}||RPW_{i}^{*})$ and $B_{i}^{*}\;=\;h(X_{i}^{*}||RPW_{i}^{*})$ and checks whether $B_{i}^{*}\overset{?}{=}B_{i}$. If it is equal, the SC sends the authentication message to the $U_{i}$.
**Step** **3:** Upon getting the message from the SC, the $U_{i}$ inputs a new password $PW_{i}^{new}$ and imprints a new biometrics $BIO_{i}^{new}$. $U_{i}$ computes $\langle R_{i}^{new},P_{i}^{new}\rangle$=$Gen(BIO_{i}^{new})$, $RPW_{i}^{new}=h(PW_{i}^{new}\left|\right|R_{i}^{new})$ and sends $\left\{RPW_{i}^{new}\right\}$ to the SC.
**Step** **4:** Upon getting the message from the $U_{i}$, the SC computes $A_{i}^{new}=X_{i}^{*}⊕h(ID_{i}^{*}||RPW_{i}^{new})$, $B_{i}^{new}=h(X_{i}^{*}||RPW_{i}^{new})$ and replaces $\left\{A_{i},B_{i}\right\}$ with $\left\{A_{i}^{new},B_{i}^{new}\right\}$.

## 6. Security Analysis

To assess secure mutual authentication of the proposed scheme, we utilize the BAN logic, which is widely accepted formal security model. Furthermore, we perform an informal security analysis to assess the safety of proposed scheme against various types of attacks.

### 6.1. Informal Security Analysis

The security of the proposed scheme is accessed utilizing an informal security analysis. Our scheme can withstand against various types of attacks, including impersonation, replay, session key disclosure attacks, and allows secure mutual authentication and anonymity.

#### 6.1.1. Impersonation Attack

When a malicious adversary $U_{MA}$ may attempt to impersonate a legitimate user, $U_{MA}$ must generate a login request message $M_{2}=h(ID_{i}\left|\right|X_{i}||RU_{i})$ correctly. However, $U_{MA}$ cannot compute it because $U_{MA}$ cannot obtain $U_{i}$’s random nonce $RU_{i}$, real identity $ID_{i}$, and secret parameter $X_{i}$. Therefore, our scheme is secure against the impersonation attack because $U_{MA}$ cannot calculate a login request message successfully.

#### 6.1.2. Replay Attack

If a malicious adversary $U_{MA}$ may attempt to impersonate legal user by resending messages transmitted in a previous session, $U_{MA}$ cannot utilize the previous messages because the CS checks whether $M_{2}^{*}\overset{?}{=}M_{2}$ and $D_{2}^{*}\overset{?}{=}D_{2}$, respectively. Furthermore, our scheme can withstand replay attack by using dynamic random nonce $RU_{i}$ and $RS_{j}$ that are changed every session. Therefore, our scheme protects against replay attack.

#### 6.1.3. Session Key Disclosure Attack

In our scheme, a malicious adversary $U_{MA}$ cannot compute session key $SK_{i}$ because $U_{MA}$ cannot obtain random nonce $RU_{i}$ and $RS_{j}$. In addition, $U_{MA}$ cannot obtain random nonce $RU_{i}$ and $RS_{j}$ without secret parameter $X_{i}$ and $SI_{j}$. Consequently, our scheme withstands the session key disclosure attack.

#### 6.1.4. Smart card Stolen Attack

According to Section 1.1, we suppose that a $U_{MA}$ can obtain a smart card and extract the data $\left\{A_{i},B_{i},Q_{i}\right\}$ stored in the smart card. However, the $U_{MA}$ cannot obtain sensitive information $ID_{i}$ and $PW_{i}$ of legitimate user because the data stored in the smart card are protected $A_{i}=X_{i}⊕h(RID_{i}||RPW_{i})$, $B_{i}=h(X_{i}||RPW_{i})$ and $Q_{i}=h(ID_{i}||PW_{i}\left|\right|R_{i})⊕RID_{i}$ by using a hash function and XOR operation.

#### 6.1.5. Mutual Authentication

In our scheme, after getting the request message $\left\{M_{1},M_{2},CID_{i},RID_{i}\right\}$ from the $U_{i}$, the control server CS checks whether $M_{2}^{*}\overset{?}{=}M_{2}$. If it is correct, CS authenticates $U_{i}$. After getting the messages $\left\{D_{1},D_{2},CSID_{j},RSID_{j}\right\}$ from cloud server $S_{j}$, the CS checks whether $D_{2}^{*}\overset{?}{=}D_{2}$. If it is equal, CS authenticates $S_{j}$. After getting the messages $\left\{M_{3},D_{3},Q_{CS}\right\}$ from the CS, the $S_{j}$ checks whether $Q_{CS}^{*}\overset{?}{=}Q_{CS}$. If it is correct, $S_{j}$ authenticates CS. After getting the messages $\left\{Q_{CU}\right\}$ from the $S_{j}$, the $U_{i}$ checks whether $Q_{CU}^{*}\overset{?}{=}Q_{CU}$. Finally, the $U_{i}$ authenticates $S_{j}$. As a result, our scheme achieve secure mutual authentication among $U_{i}$, $S_{j}$, and CS because a malicious adversary $U_{MA}$ does not know secret parameters $X_{i}$ and $SI_{j}$.

#### 6.1.6. Anonymity

A malicious adversary $U_{MA}$ cannot obtain the real identity $ID_{i}$ of legitimate user because it is masked by using hash function and XOR operation such as $CID_{i}=ID_{i}⊕h(X_{i}||RU_{i})$. In addition, the $U_{MA}$ cannot obtain secret parameter $X_{i}$ and random nonce $RU_{i}$. Consequently, our scheme provides anonymity.

### 6.2. Security Features

We shows the better security levels achieved by the proposed scheme compared with some existing schemes [8,23,24,25]. The existing schemes are insecure against various attacks, including impersonation, session key disclosure smart card stolen, and replay attacks and cannot provide mutual authentication and anonymity. Table 1 shows the analysis results of the security features.

### 6.3. BAN Logic Based Authentication Proof

We performed security analysis utilizing the BAN logic to demonstrate the secure mutual authentication of the proposed scheme. We present the BAN logic notations in Table 2. Furthermore, we define the rules, the goals, the idealized form, and the assumptions for BAN logic analysis. We prove that the proposed scheme provides secure mutual authentication among $U_{i}$, $S_{j}$ and CS.

#### 6.3.1. BAN Logic Rules

The rules of BAN logic are as below.

1. Message meaning rule:
  $$\frac{A\;|\equiv A\overset{K}{↔}B,\;A⊲{\left\{X\right\}}_{K}}{A\;\left|\equiv B\;\right|∼X}$$
2. Nonce verification rule:
  $$\frac{A\;\left|\equiv \#(X),\;A\;\right|\equiv B\;|∼X}{A\;\left|\equiv B\;\right|\equiv X}$$
3. Jurisdiction rule:
  $$\frac{A\;\left|\equiv B\;\right|⟹X,\;A\;\left|\equiv B\;\right|\equiv X}{A\;|\equiv X}$$
4. Freshness rule:
  $$\frac{A\;|\equiv \#(X)}{A\;|\equiv \#\left(X,Y\right)}$$
5. Belief rule:
  $$\frac{A\;|\equiv \left(X,Y\right)}{A\;|\equiv X.}$$

#### 6.3.2. Goals

To assess the BAN logic proof, we present the goals of the proposed scheme as below.

**Goal** **1:** $U_{i}|\equiv \left(U_{i}\overset{SK}{⟷}S_{j}\right)$
**Goal** **2:** $S_{j}|\equiv \left(U_{i}\overset{SK}{⟷}S_{j}\right)$
**Goal** **3:** $U_{i}|\equiv S_{j}|\equiv \left(U_{i}\overset{SK}{⟷}S_{j}\right)$
**Goal** **4:** $S_{j}|\equiv U_{i}|\equiv \left(U_{i}\overset{SK}{⟷}S_{j}\right)$

#### 6.3.3. Idealized Forms

To assess the BAN logic proof, we define the assumptions of the proposed scheme as below.

*Msg*_1_: $U_{i}\to S_{j}$: ${\left(RID_{i},ID_{i},RU_{i}\right)}_{X_{i}}$
*Msg*_2_: $S_{j}\to CS$: ${\left(RID_{i},ID_{i},RU_{i},RSID_{j},SID_{j},RS_{j}\right)}_{SI_{j}}$
*Msg*_3_: $CS\to S_{j}$: ${\left(ID_{i},SID_{j},RU_{i},RS_{j}\right)}_{SI_{j}}$
*Msg*_4_: $S_{j}\to U_{i}$: ${\left(ID_{i},RU_{i},RS_{j},\left(U_{i}\overset{SK}{⟷}S_{j}\right)\right)}_{X_{i}}$

#### 6.3.4. Assumptions

We present the initial assumptions to assess the BAN logic proof.

*A*_1_: $S_{j}|\equiv \left(U_{i}\overset{X_{i}}{⟷}S_{j}\right)$
*A*_2_: $S_{j}|\equiv \#\left(RU_{i}\right)$
*A*_3_: $CS|\equiv (CS\overset{SI_{j}}{⟷}S_{j})$
*A*_4_: $CS|\equiv \#(RS_{j})$
*A*_5_: $S_{j}|\equiv \left(CS\overset{SI_{j}}{⟷}S_{j}\right)$
*A*_6_: $FA|\equiv \#(RS_{j})$
*A*_7_: $U_{i}|\equiv \left(U_{i}\overset{X_{i}}{⟷}S_{j}\right)$
*A*_8_: $U_{i}|\equiv \#\left(RS_{j}\right)$
*A*_9_: $U_{i}|\equiv S_{j}\Rightarrow \left(U_{i}\overset{SK}{⟷}S_{j}\right)$
*A*_10_: $S_{j}|\equiv U_{i}\Rightarrow \left(U_{i}\overset{SK}{⟷}S_{j}\right)$

#### 6.3.5. Proof Using BAN Logic

The proof then proceeds as below.

**Step** **1:** According to $Msg_{1}$, we could get
  $$\left(S_{1}\right):S_{j}⊲{\left(RID_{i},ID_{i},RU_{i}\right)}_{X_{i}}$$
**Step** **2:** Using the message meaning rule with $S_{1}$ and $A_{1}$, we get
  $$\left(S_{2}\right):S_{j}|\equiv U_{i}|∼{\left(RID_{i},ID_{i},RU_{i}\right)}_{X_{i}}$$
**Step** **3:** From the freshness rule with $S_{2}$ and $A_{2}$, we obtain
  $$\left(S_{3}\right):S_{j}|\equiv \#{\left(RID_{i},ID_{i},RU_{i}\right)}_{X_{i}}$$
**Step** **4:** Using the nonce verification with $S_{2}$ and $S_{3}$, we get
  $$\left(S_{4}\right):S_{j}|\equiv U_{i}|\equiv {\left(RID_{i},ID_{i},RU_{i}\right)}_{X_{i}}$$
**Step** **5:** From the belief rule with $S_{4}$, we obtain
  $$\left(S_{5}\right):S_{j}|\equiv U_{i}|\equiv {\left(RU_{i}\right)}_{X_{i}}$$
**Step** **6:** According to $Msg_{2}$, we could get
  $$\left(S_{6}\right):CS⊲{\left(RID_{i},ID_{i},RU_{i},RSID_{j},SID_{j},RS_{j}\right)}_{SI_{j}}$$
**Step** **7:** Using the message meaning rule with $S_{6}$ and $A_{3}$, we get
  $$\left(S_{7}\right):CS|\equiv S_{j}|∼{\left(RID_{i},ID_{i},RU_{i},RSID_{j},SID_{j},RS_{j}\right)}_{SI_{j}}$$
**Step** **8:** From the freshness rule with $S_{7}$ and $A_{4}$, we obtain
  $$\left(S_{8}\right):CS|\equiv \#{\left(RID_{i},ID_{i},RU_{i},RSID_{j},SID_{j},RS_{j}\right)}_{SI_{j}}$$
**Step** **9:** Using the nonce verification rule with $S_{7}$ and $S_{8}$, we get
  $$\left(S_{9}\right):CS|\equiv S_{j}|\equiv {\left(RID_{i},ID_{i},RU_{i},RSID_{j},SID_{j},RS_{j}\right)}_{SI_{j}}$$
**Step** **10:** According to $Msg_{3}$, we could get
  $$\left(S_{10}\right):S_{j}⊲{\left(ID_{i},SID_{j},RU_{i},RS_{j}\right)}_{SI_{j}}$$
**Step** **11:** Using the message meaning rule with $S_{10}$ and $A_{5}$, we get
  $$\left(S_{11}\right):S_{j}|\equiv CS|∼{\left(ID_{i},SID_{j},RU_{i},RS_{j}\right)}_{SI_{j}}$$
**Step** **12:** From the freshness rule with $S_{11}$ and $A_{6}$, we obtain
  $$\left(S_{12}\right):S_{j}|\equiv \#{\left(ID_{i},SID_{j},RU_{i},RS_{j}\right)}_{SI_{j}}$$
**Step** **13:** Using the nonce verification rule with $S_{11}$ and $S_{12}$, we get
  $$\left(S_{13}\right):S_{j}|\equiv CS|\equiv {\left(ID_{i},SID_{j},RU_{i},RS_{j}\right)}_{SI_{j}}$$
**Step** **14:** According to $Msg_{4}$, we could get
  $$\left(S_{14}\right):U_{i}⊲{\left(ID_{i},RU_{i},RS_{j},\left(U_{i}\overset{SK}{⟷}S_{j}\right)\right)}_{X_{i}}$$
**Step** **15:** Using the message meaning rule with $S_{14}$ and $A_{7}$, we get
  $$\left(S_{15}\right):U_{i}|\equiv S_{j}|∼{\left(ID_{i},RU_{i},RS_{j},\left(U_{i}\overset{SK}{⟷}S_{j}\right)\right)}_{X_{i}}$$
**Step** **16:** From the freshness rule with $S_{15}$ and $A_{8}$, we obtain
  $$\left(S_{16}\right):U_{i}|\equiv \#{\left(ID_{i},RU_{i},RS_{j},\left(U_{i}\overset{SK}{⟷}S_{j}\right)\right)}_{X_{i}}$$
**Step** **17:** Using the nonce verification with $S_{15}$ and $S_{16}$, we get
  $$\left(S_{17}\right):U_{i}|\equiv S_{j}|\equiv {\left(ID_{i},RU_{i},RS_{j},\left(U_{i}\overset{SK}{⟷}S_{j}\right)\right)}_{X_{i}}$$
**Step** **18:** From the belief rule with $S_{17}$, we obtain
  $$\left(S_{18}\right):U_{i}|\equiv S_{j}|\equiv \left(U_{i}\overset{SK}{⟷}S_{j}\right)\;\;\left(\mathrm{Goal}3\right)$$
**Step** **19:** Using the jurisdiction rule with $S_{18}$ and $A_{9}$, we get
  $$\left(S_{19}\right):U_{i}|\equiv \left(U_{i}\overset{SK}{⟷}S_{j}\right)\;\;\left(\mathrm{Goal}1\right)$$
**Step** **20:** Because of $SK=h(RU_{i}||RS_{j})$, from the $S_{5}$, $S_{9}$, $S_{13}$ and $S_{17}$ we could get
  $$\left(S_{20}\right):S_{j}|\equiv U_{i}|\equiv \left(U_{i}\overset{SK}{⟷}S_{j}\right)\;\;\left(\mathrm{Goal}4\right)$$
**Step** **21:** Using the jurisdiction rule with $S_{19}$ and $A_{10}$, we obtain
  $$\left(S_{21}\right):S_{j}|\equiv \left(U_{i}\overset{SK}{⟷}S_{j}\right)\;\;\left(\mathrm{Goal}2\right)$$

Referring to Goals 1–4, we show that proposed scheme achieves secure mutual authentication among $U_{i}$, $S_{j}$ and CS.

## 7. Simulation for Security Verification with the AVISPA tool

We performed a formal security verification of the proposed scheme utilizing AVISPA simulation tool [26,27] to evaluate the safety of the authentication protocol against MITM and replay attacks, which is widely accepted for formal security analysis [28,29,30,31]. To perform AVISPA simulation tool, the environment and the session of security protocol must be implemented using the High Level Protocols Specification Language (HLPSL).

### 7.1. HLPSL Specifications

**Figure 9:**
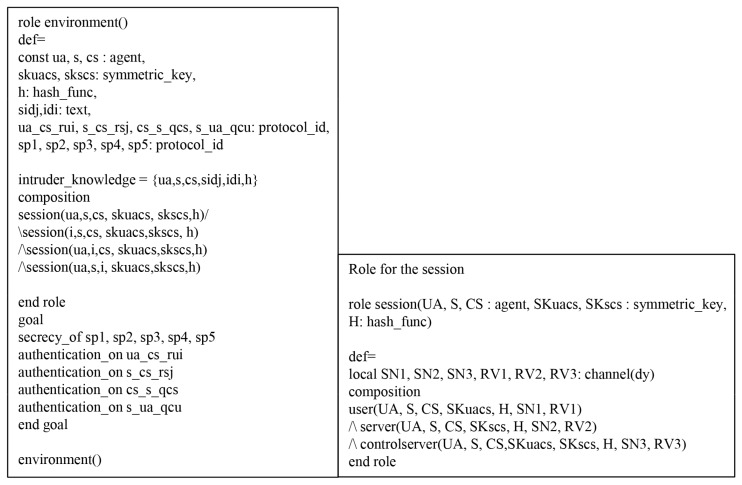
Role for environment and session in HLPSL.

**Figure 10:**
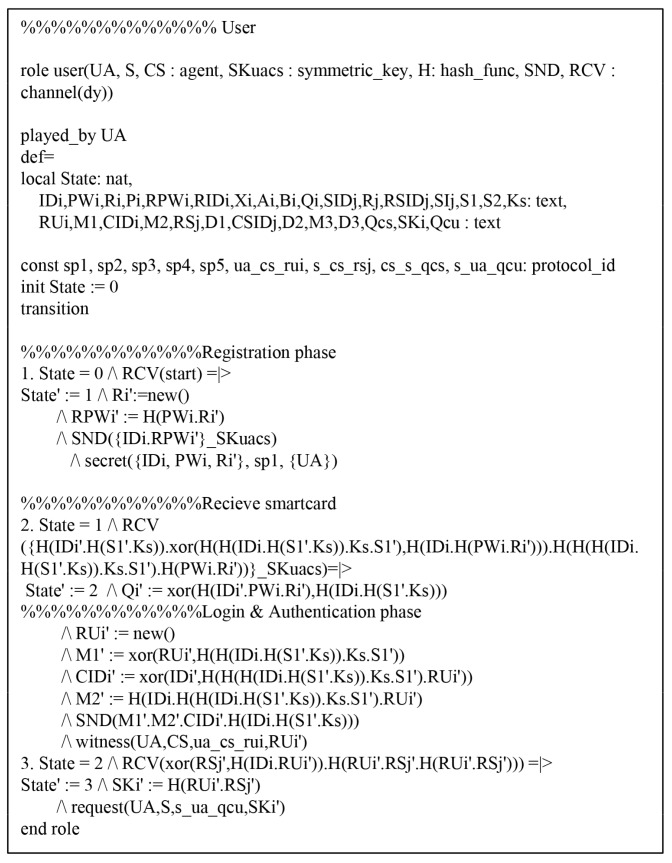
Role specification for user $U_{i}$.

**Figure 11:**
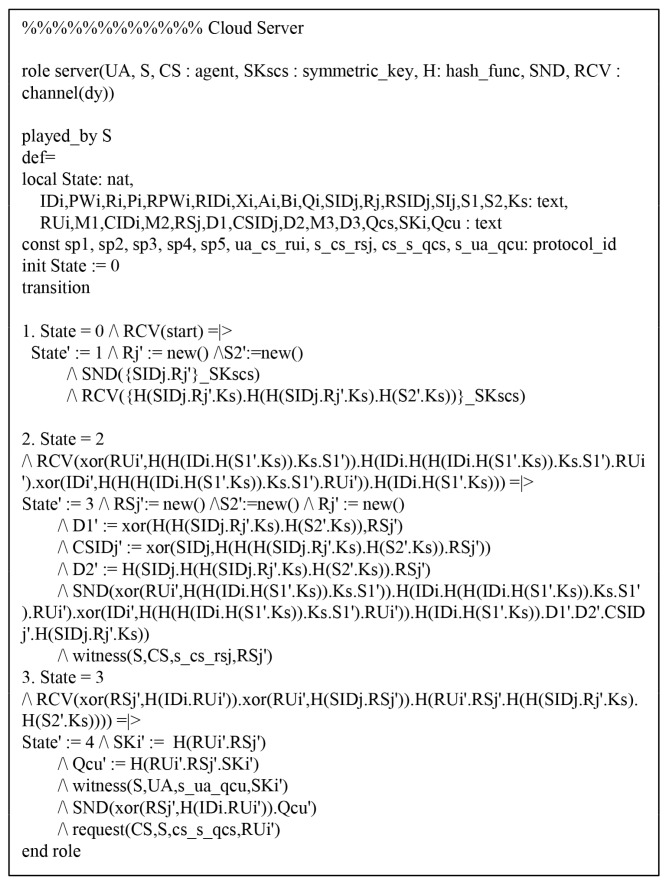
Role specification for cloud server $S_{j}$.

**Figure 12:**
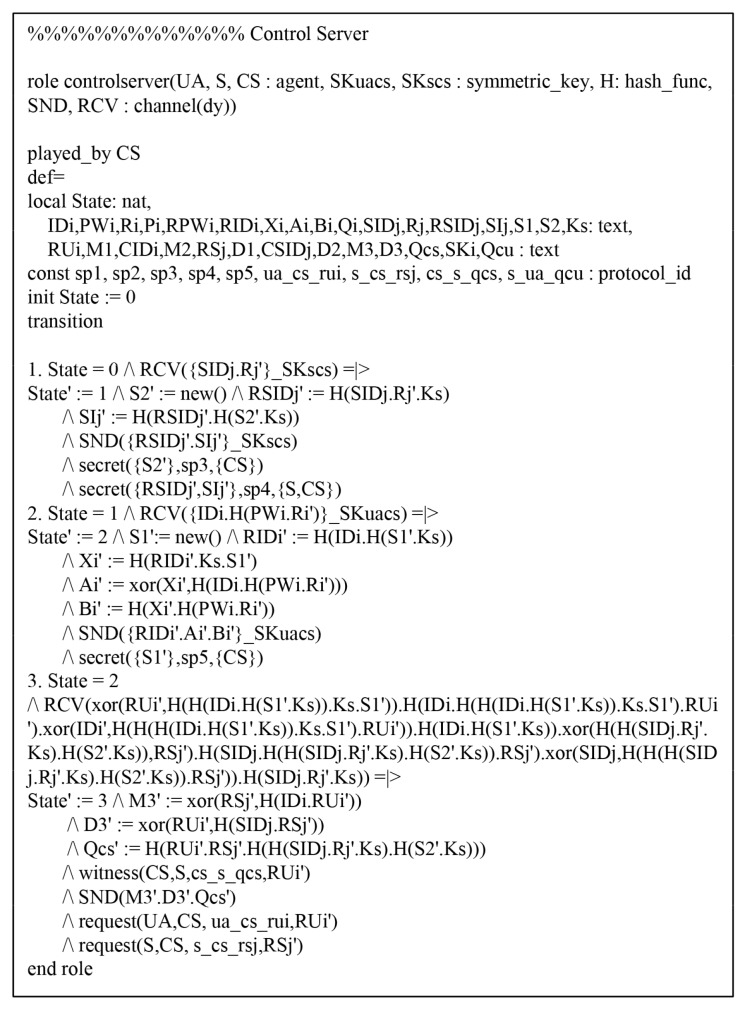
Role specification for control server CS.

We considered three basic roles: user $U_{i}$, cloud server $S_{j}$, and control server CS. Then, we present session and environment utilizing HLPSL in Figure 9, which contains the security goals. The role specifications of $U_{i}$, $S_{j}$, and CS are as shown in Figure 10, Figure 11 and Figure 12.

The $U_{i}$ receives the initial message and updates the updates the state value from 0 to 1. The $U_{i}$ then sends the registration request messages $\left\{ID_{i},RPW_{i}\right\}$ to the CS via a secure channel and receives $\left\{RID_{i},Smartcard\right\}$ from the CS. The $U_{i}$ updates the state value from 1 to 2. In the login and authentication phase, the $U_{i}$ declares $witness(UA,CS,ua\_sn\_rui,RU_{i}^{\prime })$ from the $S_{j}$, and then updates the state value from 2 to 3. Finally, the $U_{i}$ receives the authentication messages $\left\{M_{3},Q_{CU}\right\}$ from the $S_{j}$. The $U_{i}$ checks whether $Q_{CU}^{*}\overset{?}{=}Q_{CU}$. If it is valid, the $U_{i}$ authenticates the $S_{j}$ successfully. The role specification for $S_{j}$ is similarly defined.

### 7.2. AVISPA Simulation Result

**Figure 13:**
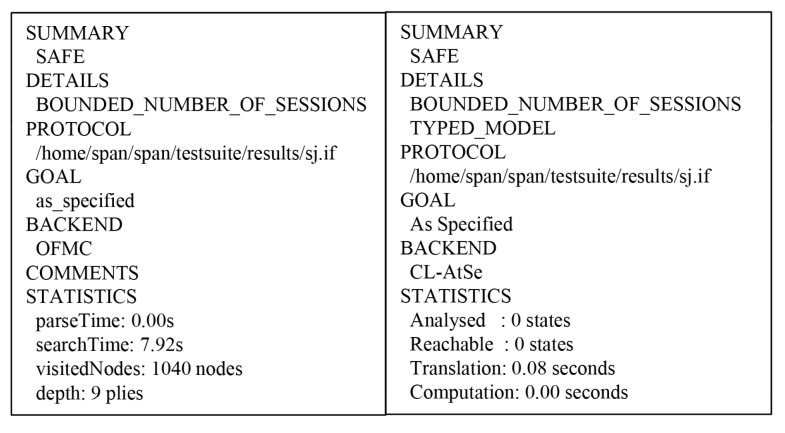
Analysis of AVISPA simulation using OFMC and CL-AtSe.

We show the AVISPA results to verify the safety of the proposed scheme using OFMC and CL-AtSe. The OFMC checks whether the proposed scheme is safe from MITM attack. In addition, the CL-AtSe demonstrates the safety of the protocol against replay attack. Consequently, Figure 13 shows that the proposed scheme is secure against MITM and replay attacks though AVISPA simulation.

## 8. Performance Analysis

We compared the computation cost, communication cost and security features of the proposed scheme with some existing schemes [8,23,24,25]. We show that the proposed scheme provides better efficiency and security features.

### 8.1. Computation Cost

We compared the computation overheads of the proposed scheme with some existing schemes [8,23,24,25]. To analyze of computation cost, we estimated using the following parameters. Table 3 shows the analysis results of computation cost and the detailed total cost are as below.

The total computation cost for the proposed scheme and Pelaez et al.’s scheme are 34$T_{h}$ and 48$T_{h}$ + 8$T_{s}$, respectively. We provide better efficiency than some existing schemes because the proposed scheme uses only hash and XOR operations. Therefore, our scheme is secure and efficient for practical IoT-based cloud computing environment.

- $T_{h}$ denotes the time for the hash function (Case 1 ≈0.00517 ms [23] and Case 2 ≈0.0000328 ms [32]).
- $T_{s}$ denotes the time for the symmetric key cryptography operation using AES algorithm (case 1 ≈0.02148 ms [23] and Case 2 ≈0.0214385 ms [32]).
- The XOR operation was not included because it is negligible compared to the other operations.

### 8.2. Communication Cost

We compared the communication overhead of the proposed scheme with some existing schemes [8,23,24,25]. In authentication phase of the proposed scheme, the transmitted messages $\left\{M_{1},M_{2},CID_{i},RID_{i}\right\}$, $\left\{M_{1},M_{2},CID_{i},RID_{i},D_{1},D_{2},CSID_{j},RSID_{j}\right\}$, $\left\{M_{3},D_{3},Q_{CS}\right\}$ and $\left\{M_{3},Q_{CU}\right\}$ require (128 + 128 + 128 + 128 = 512 bits), (128 + 128 + 128 + 128 + 128 + 128 + 128 + 128 = 1024 bits), (128 + 128 + 128 = 384 bits), and (128 + 128 = 256 bits), respectively. Table 4 shows the analysis results of communication cost. Consequently, the proposed scheme is thus more efficient than other related schemes [8,23,24,25] because the total communications cost are 2176 bits (Case 1) and 4352 bits (Case 2).

- Case 1 defines that the pseudo-identity, random nonce, timestamp, identity, password, and hash function are 128 bits, respectively.
- Case 2 defines that the pseudo-identity, random nonce, timestamp, identity, password, and hash function are 256 bits, respectively.
- The block length for symmetric encryption is 128 bits.

## 9. Conclusions

This paper shows that Pelaez et al.’s scheme does not defend various attacks such as impersonation, session key disclosure and replay attacks. Furthermore, we show that Pelaez et al.’s scheme cannot allow mutual authentication and anonymity. We propose a secure and lightweight three-factor authentication scheme for IoT in cloud computing environment to enhance the security drawbacks of Pelaez et al.’s scheme. Our scheme can withstand various types of attacks, including impersonation, session key disclosure and replay attacks, and can provide mutual authentication and anonymity. Then, we demonstrate that our scheme allows secure mutual authentication among $U_{i}$, $S_{j}$, and CS utilizing BAN logic analysis. We also performed a formal security verification analysis of the proposed scheme utilizing the AVISPA simulation tool. In addition, we compared the security features and performance of the proposed scheme with some existing schemes. We show that our scheme provides better safety and efficiency than related schemes. Therefore, our scheme can be suitable for practical IoT-based cloud computing environment because it is more secure and lightweight than the previous schemes.

## Figures and Tables

**Table 1:** Security features comparison.

Security Features	Xue et al. [24]	Amin et al. [25]	Zhou et al. [23]	Pelaez et al. [8]	Ours
Impersonation attack	×	×	×	×	∘
Smart card stolen attack	×	×	∘	×	∘
Session key disclosure attack	×	∘	×	×	∘
Replay attack	∘	∘	×	×	∘
Anonymity	×	∘	∘	×	∘
Mutual authentication	×	∘	×	×	∘

∘, preserves the security features; ×, does not preserve the security features;

**Table 2:** Notations for BAN logic.

Notation	Description
A|≡X	*A***believes** statement *X*
#X	Statement *X* is **fresh**
A⊲X	*A***sees** statement *X*
A|∼X	*A* once **said** *X*
A⇒X	*A* has got **jurisdiction** of *X*
$<X{>}_{Y}$	*X* is **combined** with *Y*
${\left\{X\right\}}_{K}$	*X* is **encrypted** under key *K*
$A\overset{K}{↔}B$	*A* and *B* may use **shared key** *K* to communicate
SK	Session key used in the current session

**Table 3:** A comparative summary: computation costs.

Schemes	User	Cloud Server	Control Server	Total	Total Cost (Case 1)	Total Cost (Case 2)
Xue et al. [24]	$12T_{h}$	$6T_{h}$	$18T_{h}$	$36T_{h}$	0.18612 ms	0.0011808 ms
Amin et al. [25]	$12T_{h}$	$4T_{h}$	$14T_{h}$	$30T_{h}$	0.1551 ms	0.000984 ms
Zhou et al. [23]	$13T_{h}$	$7T_{h}$	$23T_{h}$	$43T_{h}$	0.22231 ms	0.0014104 ms
Pelaez et al. [8]	$9T_{h}+3T_{s}$	$6T_{h}+3T_{s}$	$33T_{h}+2T_{s}$	$48T_{h}+8T_{s}$	0.42 ms	0.1730824 ms
Ours	$12T_{h}$	$6T_{h}$	$16T_{h}$	$34T_{h}$	0.17578 ms	0.0011152 ms

*T_h_*, hash function; *T_s_*, symmetric key cryptography operation using AES algorithm

**Table 4:** A comparative summary: communication costs.

Schemes	Message Length	Total Cost (Case 1)	Total Cost (Case 2)
Xue et al. [24]	30	3840 bits	7680 bits
Amin et al. [25]	27	3456 bits	6912 bits
Zhou et al. [23]	34	4352 bits	8704 bits
Pelaez et al. [8]	34	4352 bits	8704 bits
Ours	25	2176 bits	4352 bits

## References

[1] Effectively and Securely Using the Cloud Computing Paradigm (v0.25). http://csrc.nist.gov/groups/SNS/cloud-computing.

[2] Grobauer B., Walloscheck T., Stocker E. (2011). Understanding cloud computing vulnerabilities. IEEE Secur. Priv..

[3] Lamport L. (1981). Password authentication with insecure communication. Commun. ACM.

[4] Kocher P., Jaffe J., Jun B. (1999). Differential power analysis. Advances in Cryptology.

[5] Amin R., Islam S.K., Biswas G.P., Khan M.K., Leng L., Kumar N. (2016). Design of an anonymity-preserving three-factor authenticated key exchange protocol for wireless sensor networks. Comput. Netw..

[6] Jiang Q., Zeadally S., Ma J., He D. (2017). Lightweight three-factor authentication and key agreement protocol for internet-integrated wireless sensor networks. IEEE Access.

[7] Li X., Niu J., Kumari S., Wu F., Choo K.K.R. (2018). A robust biometrics based three-factor authentication scheme for global mobility networks in smart city. Future Gener. Comput. Syst..

[8] Pelaez R.M., Cruz H.T., Michel J.R., Garcia V., Mena L.J., Felix V.G., Brust A.O. (2019). An enhanced lightweight IoT-based authentication scheme in cloud computing circumstances. Sensors.

[9] Dolev D., Yao A.C. (1983). On the security of public key protocols. IEEE Trans. Inf. Theory.

[10] Park Y., Park K., Park Y. (2019). Secure user authentication scheme with novel server mutual verification for multiserver environments. J. Commun. Syst..

[11] Park K., Park Y., Das A.K., Yu S., Lee J., Park Y.H. (2019). A dynamic privacy-preserving key management protocol for V2G in social internet of things. IEEE Access.

[12] Burrows M., Abadi M., Needham R. (1990). A logic of authentication. ACM Trans. Comput. Syst..

[13] Chien H.Y., Jan J., Tseng Y.M. (2002). An efficient and practical solution to remote authentication: Smart card. Comput. Secur..

[14] Zhu J., Ma J. (2004). A new authentication scheme with anonymity for wireless environments. IEEE Trans. Cons. Elec..

[15] Lee Y., Kim S., Won D. (2010). Enhancement of two-factor authenticated key exchange protocols in public wireless LANs. Comput. Electr. Eng..

[16] Kim J., Lee D., Jeon D., Lee Y., Won D. (2014). Security anaylsis and improvements two-factor mutual authentication with key agreement in wireless sensor networks. Sensors.

[17] Wang D., Wang P. (2014). On the anonymity of two-factor authentication schemes for wireless sensor networks. Comput. Netw..

[18] Wang D., Li W., Wang P. (2018). Measuring two-factor authentication schemes for real-time data access in industrial wireless sensor networks. IEEE Trans. Indust. Inform..

[19] Wong K.H., Zheng Y., Cao J., Wang S. (2006). A dynamic user authentication scheme for wireless sensor networks. IEEE Inter. Conf. Sensor Netw. Ubiq. Trustworthy Comp..

[20] Li X., Peng J., Niu J., Wu F., Liao J., Choo K.K.R. (2018). A robust and energy efficient authentication protocol for industrial internet of things. IEEE Internet Things J..

[21] Li X., Niu J., Kumari S., Wu F., Sangaiah A., Choo K.K.R. (2018). A three-factor anonymous authentication scheme for wireless sensor networks in internet of things environments. J. Netw. Comp. Appl..

[22] Lee J., Yu S., Park K., Park Y., Park Y. (2019). Secure three-factor authentication protocol for multi-gateway IoT environments. Sensors.

[23] Zhou L., Li X., Yeh K.H., Su C., Chiu W. (2019). Lightweight IoT-based authentication scheme in cloud computing circumstance. Future Gener. Comput. Syst..

[24] Xue K., Hong P., Ma C.A. (2014). A lightweight dynamic pseudonym identity based authentication and key agreement protocol without verification tables for multi-server architecture. J. Comput. Syst. Sci..

[25] Amin R., Kumar N., Biswas G.P., Iqbal R., Chang V. (2018). A lightweight authentication protocol for IoT-enabled devices in distributed cloud computing environment. Future Gener. Comput. Syst..

[26] AVISPA Automated Validation of Internet Security Protocols and Applications. http://www.avispa-project.org/.

[27] SPAN: A Security Protocol Animator for AVISPA. http://www.avispa-project.org/.

[28] Park K., Park Y., Park Y., Reddy A.G., Das A.K. (2017). Provably secure and efficient authentication protocol for roaming service in global mobility networks. IEEE Access.

[29] Park K., Park Y., Park Y., Das A.K. (2018). 2PAKEP: Provably secure and efficient two-party authenticated key exchange protocol for mobile environment. IEEE Access.

[30] Yu S., Lee J., Lee K., Park K., Park Y. (2018). Secure authentication protocol for wireless sensor networks in vehicular communications. Sensors.

[31] Park Y., Park Y. (2016). Three-factor user authentication and key agreement using elliptic curve cryptosystem in wireless sensor networks. Sensors.

[32] Wu F., Xu L., Kumari S., Li X., Shen J., Choo K.K.R., Wazid M., Das A.K. (2017). An efficient authentication and key agreement scheme for multi-gateway wireless sensor networks in IoT deployment. J. Netw. Comput. Appl..

